# Could urinary nerve growth factor and bladder wall thickness predict the treatment outcome of children with overactive bladder?

**DOI:** 10.1590/S1677-5538.IBJU.2021.0790

**Published:** 2022-03-14

**Authors:** Adil Huseynov, Onur Telli, Perviz Haciyev, Tolga M. Okutucu, Aykut Akinci, Mete Ozkidik, Imge Erguder, Suat Fitoz, Berk Burgu, Tarkan Soygur

**Affiliations:** 1 Ankara University School of Medicine Department of Pediatric Urology Ankara Turkey Department of Pediatric Urology, Ankara University, School of Medicine, Ankara, Turkey; 2 Kartal Dr. Lutfi Kirdar Training and Research Hospital Clinic of Urology Istanbul Turkey Clinic of Urology, Kartal Dr. Lutfi Kirdar Training and Research Hospital, Istanbul, Turkey; 3 Ankara University School of Medicine Department of Medical Biochemistry Ankara Turkey Department of Medical Biochemistry, Ankara University, School of Medicine, Ankara, Turkey; 4 Ankara University School of Medicine Department of Radiology Ankara Turkey Department of Radiology, Ankara University, School of Medicine, Ankara, Turkey

**Keywords:** Urinary Bladder, Overactive, Lower Urinary Tract Symptoms, Urinary Bladder, Neurogenic

## Abstract

**Objective:**

Bladder wall thickness (BWTh) measurements and Nerve Growth Factor (NGF) /creatinine (Cr) values, as noninvasive tools, were found to predict daytime voiding problems in children with overactive bladder (OAB). The goal of this research was to examine if bladder wall thickness together with urine NGF/Cr could be a clinical utility in treatment outcome of OAB in children.

**Patients and Methods:**

A total of 60 children with OAB, (Group 1; n=40) and healthy normal controls (Group 2; n=20), aged 6-14 years old were involved in this prospective study. Children were evaluated with detailed history and physical examination, including neurologic examination, and were asked to complete a self-reported questionnaire and a 3-day bladder diary with the aid of their parents. Uroflowmetry was performed in all cases. Urinary nerve growth factor levels were measured by the ELISA and BWTh was measured trans-abdominally by one uro-radiologist specialized in pediatric ultrasonography. Urinary NGF levels were normalized by urinary creatinine levels and compared among all subgroups. Children with OAB received urotherapy as first line treatment at least for three months. 18 children refractory to urotherapy received anticholinergic therapy defined as group 3.

**Results:**

The median age of the study group was 10 (range 6 to 16). After urotherapy, 22 children had similar BWTh and NGF/Cr values compared to controls. (2.75 ± 1.15; 2.40 ± 1.00 mm; p=0.86 and 1.02 ± 0.10; 0.78 ± 0.15; p=0.12, respectively). After anticholinergic treatment, BWTh levels (2.25 ± 0.90; 2.40 ± 1.00 mm; p=0.94) and NGF/Cr values (0.95 ± 0.10; 0.78 ± 0.15; p=0.42, respectively) had no significantly difference compared to controls (Group 2).

In receiver operating characteristic analysis, bladder wall thickness was found to have sensitivity of 85% and specificity of 84.2% (3,20 AUC, 913; 95 %) and NGF/Cr had sensitivity of 90% and specificity of 92.1% (1,595; AUC, 947; 95 %) in predicting treatment outcome in children with OAB.

**Conclusions:**

Bladder wall thickness measurements and NGF/Cr values, as noninvasive tools, could guide outcomes in the treatment of children with overactive bladder.

## INTRODUCTION

Daytime lower urinary tract condition (dLUTC) is a complex term that consists of heterogeneity of symptoms of LUT dysfunction (1). In the absence of other (e.g. neurological) diseases, overactive bladder is a symptom complex comprising of uncomfortable stored urine symptoms such as urinary frequency, urgency, and nocturia (2). The main diagnostic tools for overactive bladder (OAB) are a detailed history, physical exam, urinalysis (to rule out infection and microscopic hematuria), a post-void residual measured by ultrasound, and a frequency-volume chart (which can highlight fluid intake, average and maximum bladder volumes, and timing of voids). When the diagnosis is unknown or there is a high suspicion for another ailment, more advanced diagnostic modalities such as urodynamics, cystoscopy, or upper tract imaging are required (1, 2). However, daytime symptoms may overlap between conditions such as an OAB or non-monosymptomatic nocturnal enuresis accompanied by urinary frequency and nocturia with or without urinary incontinence (3). Since border cases are common, clinical examination of dLUTC including fluid intake, frequency volume chart and uroflowmetry could be inaccurate in children. In addition, urodynamic studies (UD) are not routinely used to measure volumes, pressures and flows due to the disadvantage of patient discomfort unless a suspected neuropathic bladder (4). Thus, instead of UD, simple and less invasive tests that should play a crucial role in diagnosis and treatment outcome of dLUTC are needed. Recent studies considered urinary nerve growth factor (NGF) as a biomarker for sensory urgency, detrusor overactivity and OAB by the activation of sensory receptors of urothelium and smooth muscle resulting in an increase in urinary NGF (5-7). Moreover, bladder wall thickness (BWTh) has been reported to be preferable to the UD study and could be replaced as a diagnostic parameter in children with OAB (8-10). In our previous report, we found that, BWTh and NGF /creatinine (Cr) values, as noninvasive tools, were found to predict daytime voiding problems in children (11). We hypothesized that, increased levels of BWTh and NGF /creatinine (Cr) values might decrease as a result of treatment. In this study, we aimed to evaluate the value of BWTh together with urine NGF/Cr in treatment outcome of OAB.

## MATERIAL AND METHODS

### Patients and Study Design

**Figure 1:**
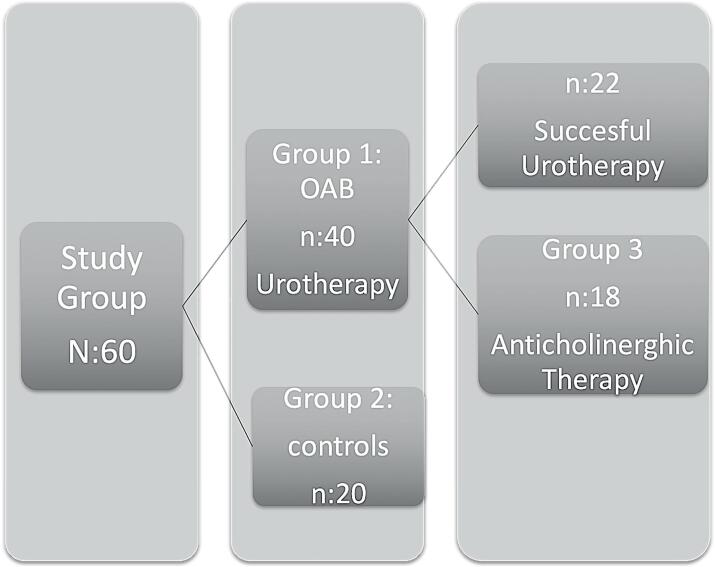
Flow chart of the study.

Between April 2015 and January 2017, 60 children aged 6 to 16 years old referred to the department of pediatric urology were prospectively recruited in the study with the agreement of the institutional review board (IRB 2015/650/15). Informed consent was obtained from all of the parents. There were three groups in the study. Group 1 included children with dLUTC (n = 40) and group 2 from pediatric urology clinic without OAB or any other urinary symptoms as controls. (n=20). Children with OAB in group 1 received urotherapy as first line treatment at least 3 months. 18 children refractory to urotherapy received anticholinergic therapy at least 3 months defined as group 3 (Figure-1).

At initial presentation, past history was taken and neurologic examination was performed. With the aid of parents, 3-day bladder and diary dysfunctional voiding symptom score were asked to be completed. It was reported that a score of 8.5 may be an optimum threshold score to determine whether the subject has clinically significant wetting and functional voiding symptoms, with a sensitivity of 90% and a specificity of 90% (12). The presence of daytime voiding symptoms as urgency, urge-incontinence, incontinence, holding maneuvers, frequency and fluid intake were recorded. Uroflowmetry was also performed in all children. Abnormal uroflowmetry patterns (plateau, staccato-shaped or interrupted pattern), children with lower urinary tract obstruction, renal dysfunction, developmental disorder, urinary tract infection documented in previous three months, congenital urothelial malformation were excluded. Urotherapy was recognized as a behavioral intervention to reduce symptoms by establishing a functional voiding behavior. In any case, timed voiding every three to four hours during daytime, was instituted early on with a sufficient period of time to achieve complete bladder emptying. In terms of voiding technique, children were asked to void with their legs spread apart and a footstool was advised to be used for the child's heals to touch the ground if the toilet does not have a proper height. Fluid intake was advised regularly during the day however towards bedtime suggested to be minimalized. Beverages that can trigger urgency and frequency symptoms, such as those containing caffeine, chocolate or citrus, and carbonated beverages were advised to be avoided. Bowel management staying well hydrated and having a high-fiber diet was planned as a part of urotherapy after obtaining Bristol stool scale (13). A 50% improvement rate of frequency and urgency symptoms was accepted as success as reported previously after simple behavioral therapy (14). Although the patients received at least three months urotherapy, less than 50% improvement in OAB symptoms was accepted as refractory to therapy. Patients received oxybutynin suspension with a recommended daily dose of 0.3–0.6 mg/kg to the maximum dose of 15 mg/kg/day. International Children's Continence Society was followed for the definitions of dLUTC (1).

### Bladder Wall Thickness Measurement

Ultrasonography (US) with a high frequency (7.5 MHz) linear probe was performed by one pediatric radiologist in supine position from suprapubic region. Children notice to void was (first urge) accepted as the time of BWTh measurement in each patient. The technique describes the distance between two hyperechogenic lines, which represent the adventitia and mucosa and/or submucosal tissue as BWTh (10). The average of three layers as anterior, posterior and lateral, divided by three was accepted as the mean BWTh.

### Urinary Marker Analysis

Urine samples were collected in a culture sterile period. The samples were put on ice immediately and centrifuged at 3000 rpm for 10min at 4°C. The supernatant was separated into aliquots in 1.5 mL tubes and stored at −80°C freezer. The total urinary NGF level was further normalized by the concentration of urinary creatinine (Cr) level (NGF/Cr level). Urinary Cr levels were measured after 3 mL urine was taken to normalize NGF levels. Emax^®^ ImmunoAssay System (Promega, Madison, WI, USA) was used to determine Urinary NGF concentration. Assays were validated for urine measurements according to the manufacturer's instructions. The detailed procedure was described in our previous report (11).

#### Statistical Analysis

Statistical analyses were performed with SPSS 25.0, and the statistical significance was set as P <0.05. Statistical analysis was performed using the nonparametric Mann-Whitney U test. Kruskal-Wallis and Wilcoxon signed-rank tests were used to analyze the groups. A receiver operating characteristic (ROC) curve was used to determine a cut-off value of BWTh and NGF/Cr rate in the treatment outcome of OAB.

## RESULTS

A total of 60 children consisting of 44 girls and 16 boys with a median age of 10 (6-16) years were enrolled to the study. There were no significant differences between groups according to gender, age and functional voided volumes between groups (p > 0.05). Table-1 lists the demographic and micturition data of each group. The voiding symptom score before the treatment, was 14.6 ± 2.4 in the group 1 and this value was 4.5 ± 2.5 in controls (Group 2).

**Table 1:** Patient's characteristics of treatment groups (Group 1 and Group 3) and controls (Group 2).

		Group 1 (n=40)	Group 2 (n=20)	Group 3 (n=18)	P
Median age (min-max, years)		9 (6-16)	10 (7-15)	10 (6-15)	0.523
Sex (M: F)		14/26	8/12	9/9	0.326
**Mean ± SD daytime voiding**					
	Voids/day	7.2 ± 1.0	4.8 ± 1.2	8.0 ± 0.8	0.038
	MVVw (mL)	311 ± 112	344 ± 98	291 ± 134	0.188
**Daytime symptoms**					
	Frequency (%)	30 (60.0)	-	13(72.2)	
	Urgency (%)	22 (55.0)		16 (88.8)	
	Daytime incontinence (%)	18 (45.0)		7 (38.8)	
	Constipation/encopresis (%)	10 (25.0)		8 (44.4)	
**Symptom Score (Pre-Treatment)**		14.6±2.4	4.5±2.5	18.4±2	

The mean bladder wall thickness was significantly higher in group 1 compared to group 2 (5.10 ± 0.70 mm, 2.40 ± 1.00 mm; p<0.001). Urinary levels of nerve growth factor corrected to urine creatine were significantly higher in group 1 compared to group 2 (2.75 ± 1.15 vs.0.78 ± 0.15; p<0.001). After urotherapy, 22 children had similar BWTh and NGF/Cr values compared to controls (2.75 ± 1.15; 2.40 ± 1.00 mm; p=0.86 and 1.02 ± 0.10; 0.78 ± 0.15; p=0.12, respectively). The voiding symptom score, which was 14.6 ± 2.4 before treatment in group 1, decreased to 6.2±1.6 after the treatment in 22 children. Before anticholinergic therapy, group 3 (refractor to urotherapy, n=18) had significantly higher BWTh (4.90 ± 0.70 mm, 2.40 ± 1.00 mm; p<0.001) and NGF/Cr values (2.55 ± 1.05 vs.0.78 ± 0.15; p<0.001) compared to controls (group 2). After anticholinergic treatment, BWTh levels (2.25 ± 0.90; 2.40 ± 1.00 mm; p=0.94) and NGF/Cr values (0.95 ± 0.10; 0.78 ± 0.15; p=0.42, respectively) had no significantly difference compared to controls (Table-2). The voiding symptom score, which was 18.4 ± 2 before anticholinergic treatment in group 3, decreased to 6.8±1.4 after the treatment.

**Table 2:** Comparison of bladder wall thickness (BWTh) and NGF/Cr levels between groups.

	Groups	BWTh (mm, mean ± SD)	P value	NGF/Cr (mean ± SD)	P value
Before Urotherapy	Group 1 vs. Group 2	5.10 ± 0.70 vs. 2.40 ± 1.00	p<0.001	2.75 ± 1.15 vs. 0.78 ± 0.15	p<0.001
After Urotherapy-Before anticholinergic therapy	Group 3 vs. Group 2	4.90 ± 0.70 vs. 2.40 ± 1.00	p<0.001	2.55 ± 1.05 vs. 0.78 ± 0.15	p<0.001
After anticholinergic therapy	Group 3 vs. Group 2	2.25 ± 0.90 vs. 2.40 ± 1.00	p=0.94	0.95 ± 0.10 vs. 0.78 ± 0.15	p=0.42

p<0.001 between patients and controls for all values compared (Mann-Whitney U or Wilcoxon test, 95% CI).

**Group 1** - Children with overactive bladder; **Group 2** - Healthy normal controls; **Group 3** - Children with overactive bladder refractory to urotherapy

**Figure 2:**
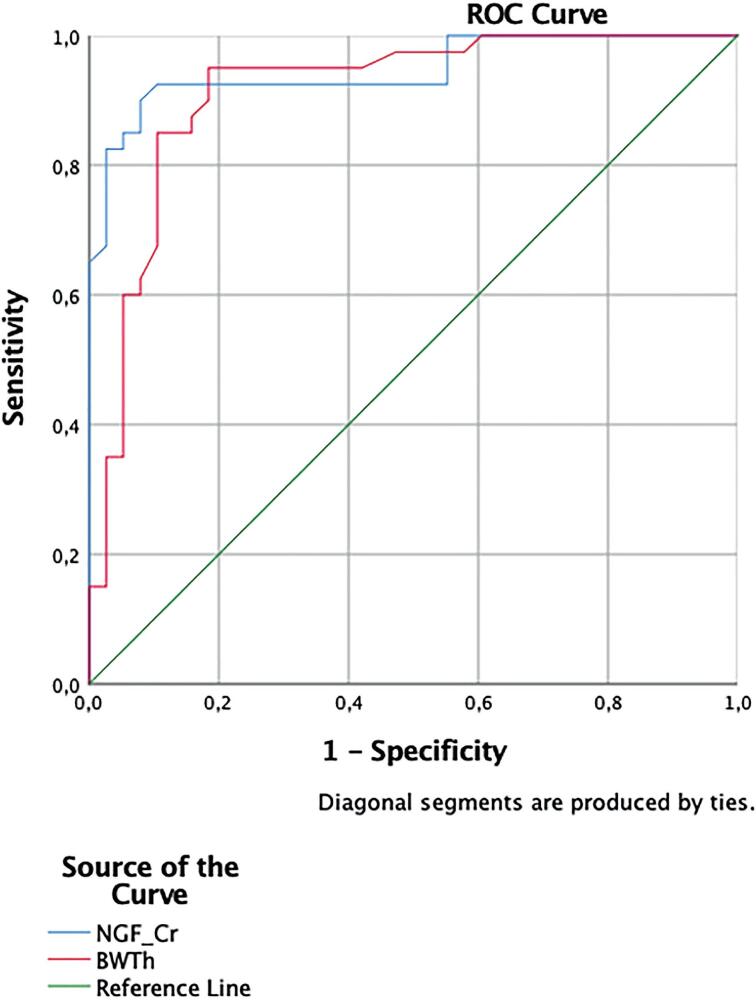
Sensitivity and specificity of BWTh and NGF/Cr in predicting treatment outcome of dLUTC (BWTh sensitivity 85%, specificity 84.2%, cutoff 3.20, AUC 913; 95%, p<0.001; NGF/Cr sensitivity 90.5%, specificity 92.1%, cut-off 1.595, AUC 947; 95%, p<0.001).

In receiver operating characteristic analysis, bladder wall thickness was found to have sensitivity of 85% and specificity of 84.2% (3.20 AUC 0.913; 95 %, p<0.001) and NGF/Cr had sensitivity of 90.5% and specificity of 92.1% (1,595; AUC, 947; 95 %, p<0.001) in predicting treatment outcome of OAB in children refractory to urotherapy (Figure-2).

## DISCUSSION

Urinary NGF secreted by urothelium and smooth muscles was considered as a new biomarker of lower urinary tract disorders such as interstitial cystitis, OAB, bladder outlet obstructions and chronic prostatitis (15, 16). NGF may play a role in urinary bladder dysfunction through promoting inflammation, as well as morphological and functional alterations in the sensory and sympathetic neurons that innervate the bladder. There is a disruption in the autonomic balance between the sympathetic and parasympathetic neural systems in patients with OAB, as well as less sympathetic activity at the post-voiding instant (17). Several studies have advocated the association between increased levels of NGF in urine and OAB in adult population (6, 11, 16). However, few studies focused on the link between OAB and NGF in children (7, 18). US has been reported as a non-invasive and useful tool in lower urinary tract dysfunctions by measurement of detrusor wall thickness. Frequent detrusor contractions cause hypertrophy of detrusor muscles and therefore thickened detrusor wall in overactivity of bladder in OAB produces more NGF and measurement of BWTH.

Following our first report that clarifies a clinically useful tool with urinary NGF and BWTH in diagnosis of daytime voiding problems in children, the present study was designed to investigate the therapeutic efficacy of BWTh and urinary NGF in children with OAB which is one of the possible condition of dLUTC (11).

dLUTC has a wide range in diagnosis since criteria are mostly variable. Cases can be presented as OAB, non-monosymptomatic nocturnal enuresis or signs of detrusor overactivity. In our study, urinary NGF/Cr levels and BWTh measurements were significantly higher in patients with OAB (group 1) compared to the control (group 2). Children with OAB received urotherapy and after therapy 22 of 40 children had similar BWTh and NGF/Cr results with controls. Standard urotherapy is accepted as the first line treatment for treating OAB in children and adolescents (1). A recent meta-analysis reported standard urotherapy is an effective treatment of daytime urinary incontinence compared to a spontaneous remission rate of 15.40% per year. About 56 of 100 patients was found to be recovered after being treated with SU, whereas only 15 out of 100 remit spontaneously (19).

Eighteen children (Group 3) had significantly higher BWTh and NGF/Cr although they received urotherapy. Daytime symptoms suggestive of an overactive bladder may increase bladder wall thickness, and thus NGF levels may increase in the lower urinary system after denervation, inflammation and mechanical tension. NGF and bladder wall thickness have a similar pattern. When the BWTh increases, there is a tendency that the urine NGF increases (11). After anticholinergic treatment, the thickened bladder wall and therefore NGF/Cr decreased (similar with controls) in response to the anticholinergic treatment. As put forward by Fukui et al., OAB symptoms improved after urotherapy and anticholinergic therapy in 26 (74%) of 35 children, whereas urinary NGF/Cr levels remains significantly higher in refractory group than in improved group (7). Otherwise, Liu and coworkers found that adult patients with overactive bladder who refract to anticholinergic therapy, present with high serum NGF and urinary NGF/Cr levels, and they remain high after subsequent other anticholinergic therapies (20). Surprisingly, the current findings could aid in the treatment of a carefully selected patient population suffering from severe refractory OAB symptoms due to high urine NGF, who could be administered sacral neuromodulation as a third-line treatment (21, 22). According to video-urodynamic studies, Fukui et al. reported three cases of bladder outlet obstruction in children with OAB who were refractory to urotherapy and anticholinergic medication (two urethral stenosis and one detrusor sphincter dyssynergia). Although this is likely in only a small number of cases, it is crucial to remember that children with high urine NGF/Cr levels before treatment, as well as those who don't respond to treatment, may have bladder outlet obstruction. More research is needed to determine whether video-urodynamic tests should be used to detect urethral stenosis or bladder outlet obstruction in children with OAB who are resistant to treatment and have elevated urine NGF/Cr levels prior to treatment (7).

Our work clearly has some limitations. First one is given the small number of patients and controls with only one urinary NGF and BWTH measurement could affect the results. Secondly, serum NGF levels was not carried out in this study and could have important implications. Inconsistent fluid intake in children before US or differences in resolution of the ultrasound probe may affect the BWTh measurements. The BWTh clearly depends on degree of distension. It will be maximal when the bladder is empty and at its minimum when the bladder is at maximum actual bladder capacity. Moreover, these parameters are only meaningful when referenced to age-specific normal (since a normal bladder is thinner in a younger patient than an older patient). Measuring BWTh at intermediate volumes (between empty and full) might be useful if the same relative volume was used in all cases, for example 50% or 70% of maximal capacity Multiple measurements should be considered to minimalize these effects. Lastly, BWTH and therefore NGF could be affected in bladder outlet obstructions that most of avaible data on detrusor wall thickness are from those pathologies. Thus, exclusion criteria's in diagnosis of dLUTC have a crucial point. Despite this, we believe our work could be a framework for diagnosis and treatment outcome of daytime lower urinary tract conditions by urinary NGF and BWTH.

## CONCLUSION

Bladder wall thickness and urinary levels of NGF are increased in children with OAB. To our knowledge, the present study is the first to evaluate the combination of urinary NGF and bladder wall thickness in children with OAB, which may serve as a noninvasive tool for outcome of treatment in OAB. Further studies including larger number of patients would be of great interest.
